# Young Children’s Representation of Locations in a Series: A Front-Back Representation or an Ordinal Representation?

**DOI:** 10.3389/fpsyg.2020.01327

**Published:** 2020-06-30

**Authors:** Qingfen Hu, Yuejia Fu, Yi Shao

**Affiliations:** ^1^ Institute of Developmental Psychology, Beijing Normal University, Beijing, China; ^2^ Faculty of Psychology, Beijing Normal University, Beijing, China; ^3^ Department of Psychology, Oklahoma City University, Oklahoma City, OK, United States

**Keywords:** front-back array, ordinal relation, location encoding, intrinsic reference frame, preschool children

## Abstract

Previous research has found that 3–5-year-old children could encode and retrieve a target location in a two-location series. In a paradigm of running two symmetrical railcars on a circular track, the study suggested that children used front-back array to help coding. That is, children at this age code the railcar running in the front of another as “the location in the front” and the railcar running in the back of another as “the location in the back.” However, the children’s success could be attributed to an alternative interpretation; using an ordinal representation to encode the location in front as the first with the other as the second. The current study used a four-location series to examine the children’s mental representation. Three- to five-year-old children participated in a hide-and-seek game to remember a target location out of four locations that moved in a series. The results showed salient individual differences in children’s representation, and their performance improved as the representation progressed. An ordinal representation supported the precise encoding of each location, while a vague front-back representation and a clearer front-middle-back representation led to different performance.

## Introduction

Three spatial reference frames are available in a human’s encoding and retrieving process: egocentric reference frame, in which locations are encoded with respect to the individuals themselves; allocentric reference frame, in which locations are encoded with respect to the external environment; and intrinsic reference frame, in which locations are encoded with respect to other associated locations (Negen and Nardini, 2015). The intrinsic reference frame has received relatively less attention in the field of children’s spatial cognition compared to the other two (e.g., Acredolo, 1978; Landau and Spelke, 1988; Lepecq and Lafaite, 1989; Hermer and Spelke, 1994, 1996; Huttenlocher et al., 1994; Lourenco and Frick, 2013). Prior studies have found that children could not use complex intrinsic spatial relations involving several arrayed locations until age 5 (Nardini et al., 2006; Negen and Nardini, 2015). A recent study, in contrast, focused on the age onset of using simple spatial relations in the intrinsic layout and found that children were able to use front-back array to code locations at 3–4 years of age (Hu et al., 2017).

In the study of Hu et al. (2017), children were presented with two symmetrical railcars running on a circular track. Their task was to remember one of the railcars as the hiding place and retrieve this target location after the railcars had run for a period of time. According to the authors, the direction of the movement of the railcars provided a motion-induced front-back array. Therefore, if children could retrieve the target location in this condition, but not at a condition where only mental rotation could be used (with the motion cue unavailable to provide a front-back array), this would indicate that the children successfully used the intrinsic front-back array to represent spatial locations. This study might remind readers of the famous train task by Piaget (1946/1969). For examining children’s understanding of the relation between speed, time, and distance, two trains were set to run in two parallel tracks, usually at different speeds or for different durations. The topic and design in Hu et al. ’s study is different from Piaget’s. In their experiment, the railcars ran at the same speed and time. This ensured that their relative positions remained constant.

Although the results did support their hypothesis, Hu et al. (2017) failed to exclude an alternative explanation in their study. In addition to encoding the two railcars as one in the front and the other in the back, children could also encode them using ordinal labels. That is, they could encode the front railcar as “the first one” and the back one as “the second one.” In the original design with only two locations, it was impossible to disentangle these two possibilities and to identify the corresponding representation used by children. In the present study, we aimed to address this question and to acquire further information about children’s representation.

Order is a helpful and frequently used spatial cue for coding locations. In Piaget’s view, order is a topological spatial relation that is “established when two neighboring though separate elements are ranged one before another (Piaget and Inhelder, 1956/1997, p. 7).”

Although previously, order was rarely studied as a spatial cue, some studies have provided evidence that children can understand order and ordinal labels. First, infants in their first years remembered serial of actions or events in the correct order (e.g., Merriman et al., 1997; Gulya et al., 1999; Baldwin et al., 2001; Bauer et al., 2001). Second, although infants have shown high sensitivity to serial-order, the ordinal numerals are rather difficult for children several years older (Fisher and Beckey, 1990; Colomé and Noёl, 2012). Children could not identify the Nth item in a series of seven objects until 5–6 years of age (Miller et al., 2000). Third, between 4 and 5 years, children’s performance using ordinal labels in a search task improved dramatically (Miller et al., 2015), indicating that the ordinal labels helped to encode locations.

The present study extended the study by Hu et al. (2017) by increasing the possible locations from two to four. Children viewed four railcars running one after another on a circular route. They also saw a coin being placed into one railcar and were asked to retrieve the coin while the apparatus had been in motion for some time. This design, involving four alternative locations, provided an opportunity to explore whether children would encode the target location in a front-back representation or in an ordinal representation. Children’s representations would manifest in their performance. If children used a front-back representation and encoded the first location as “in the front” and the last as “in the back,” their accuracies for these two locations would be higher than the two middle locations. If children used a front-back representation but encoded the first half as “in the front” and the last half as “in the back,” they would be able to discriminate the first two locations from the last two, but not discriminate the two locations in the same half from each other. In contrast, if children used an ordinal representation, their performance would be equal for all four locations. Hu et al. (2017) found an evident developmental change between ages 3 and 5. We thus tested children aged 3–5 years. We also expected a developmental change in children’s representation of encoding locations in series.

## Materials and Methods

### Participants

The participants were 29 3-year-olds (*M* = 3;6, range = 3;0–3;11, 12 girls), 22 4-year-olds (*M* = 4;5, range = 4;0–4;11, 10 girls), and 26 5-year-olds (*M* = 5;5, range = 5;0–5;11, 16 girls) from a local kindergarten in Beijing, China. An additional 4-year-old and two 5-year-olds participated but were excluded either because they failed to complete the task or could not understand the task. The protocol of the study was approved by the Institutional Review Board of the Faculty of Psychology, Beijing Normal University. Written informed consent was provided by the parents and oral consent was provided by each child before the experiment.

### Apparatus

**Figure 1:**
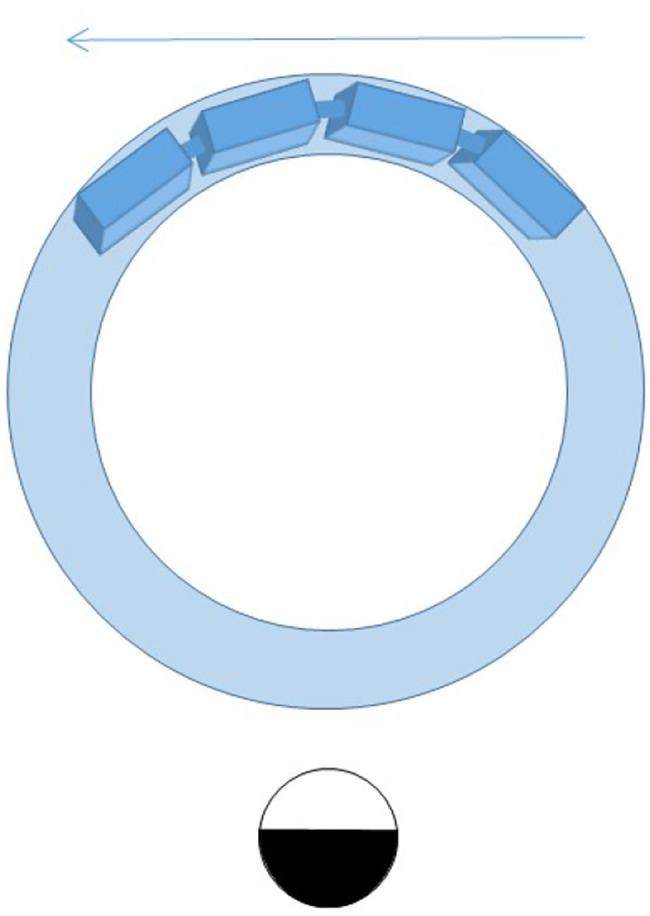
Apparatus used in the experiment.

A circular wooden rail (45 cm in diameter) was placed on featureless floor. Four identical railcars (9.6 cm × 3 cm × 6 cm), linked together, were running (13 s/revolution) on the rail, propelled by an electrical engine. The railcars were symmetrical with no cues to discriminate their front (see Figure 1). When the railcars were running, a low noise was made, which thus provided a distinct cue to indicate that they were running.

### Procedure

A female experimenter tested the children individually in a room in the kindergarten. During the warm-up phase, the participants were shown that the railcars were running on the rail and told that the railcars would run all the time. Then, the experimenter invited participants to play a hide-and-seek game. The experimenter put a coin in a predetermined railcar, drawing the participant’s attention to it. After 1 or 2 s, the experimenter helped the participant to close his/her eyes and walked the participant approximately one-quarter circle around the rail. During the walk, the experimenter blocked the participant’s view of the apparatus. After the participant was stopped, he/she was asked to open his/her eyes and point to the railcar in which he/she thought the coin would be. The child’s first choice was recorded. The experimenter then took out the coin and began the next trial.

### Design

Each participant completed four trials, in which the coin was hidden in each railcar once. The order of hiding positions was counterbalanced across all participants.

### Data Analyses

Preliminary analysis showed no significant gender effect for accuracy. Therefore, data were collapsed across gender in subsequent analyses. We conducted a 3 (Age) × 4 (Hidden Position) mixed ANOVA, with Hidden Position as a within-subject variable, Age as a between-subject variable, and accuracy as dependent variable, to test potential age difference, difficulties of each hidden position, and the potential interaction. To further analyze the developmental trajectory, the participants were divided into four groups according to their performance. Then, we conducted four repeated measure tests with choice as dependent variable for each group, in order to test the distribution of participants’ choices for each item. To better describe the participants’ performance, we also conducted one-sample *t*-tests to compare each of their choices to chance level (0.25).

## Results

**Figure 2:**
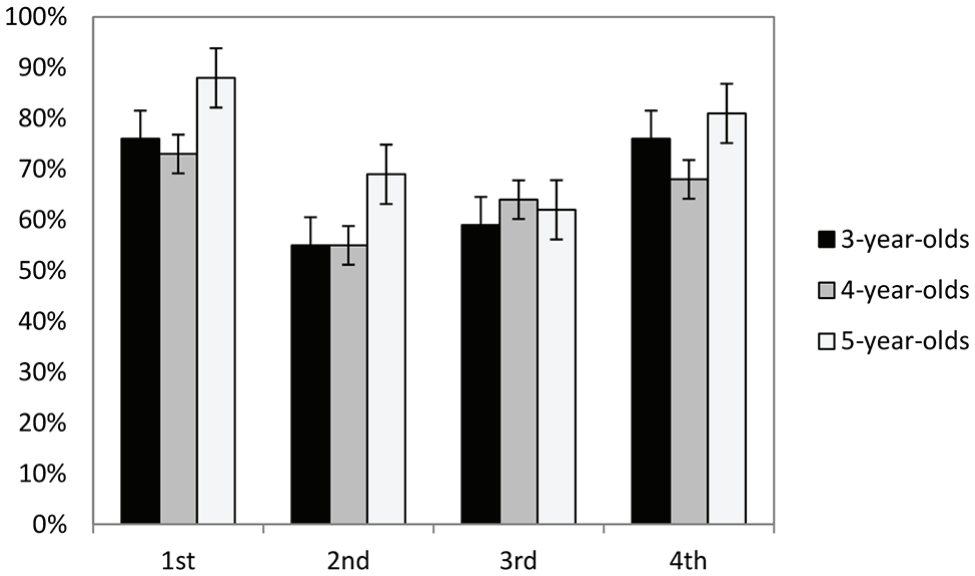
Percentage of correct choices for each age group in different hidden locations.

The 3 (Age) × 4 (Hidden Position) mixed ANOVA revealed the main effect of Hidden Position, *F*(3, 222) = 3.93, *p* < 0.01, and *η_p_* ^2^ = 0.05. The main effect of Age was not significant, *F*(2, 74) = 1.01, *p* = 0.37. The interaction of Age × Hidden Position was also not significant, *F*(6, 222) = 0.29, *p* = 0.94. Multiple comparison (Bonferroni) showed that when the coin was hidden in the first or last railcar, the children’s performance was better than when the coin was hidden in the two middle railcars, *ps* < 0.05 (see Figure 2).

To further analyze their developmental trajectory, the participants were divided into four groups: (1) Perfect: participants who searched correctly in all the four trials, (2) Near Perfect: participants who searched correctly three times in four trials, (3) Half Correct: participants who searched correctly two times in four trials, and (4) At Chance: participants who searched correctly less than two times in four trials. The age distribution of each group is presented in Table 1. The groups’ performance did not appear to depend on age, *χ* ^2^ (6, *N* = 77) = 7.31, *p* = 0.23. The age difference was also tested using age in months. A one-way ANOVA with group status as independent variable and month age as dependent variable found no significant effect, *F*(3, 73) = 0.98, *p* = 0.41.

**Table 1:** Age distributions in each group.

	3-year-olds	4-year-olds	5-year-olds	Total	Month age
Perfect	7	5	11	23 (29.87%)	55.87
Near Perfect	12	6	8	26 (33.77%)	52.92
Half Correct	4	8	4	16 (20.78%)	51.94
At Chance	6	3	3	12 (15.58%)	50.17

**Figure 3:**
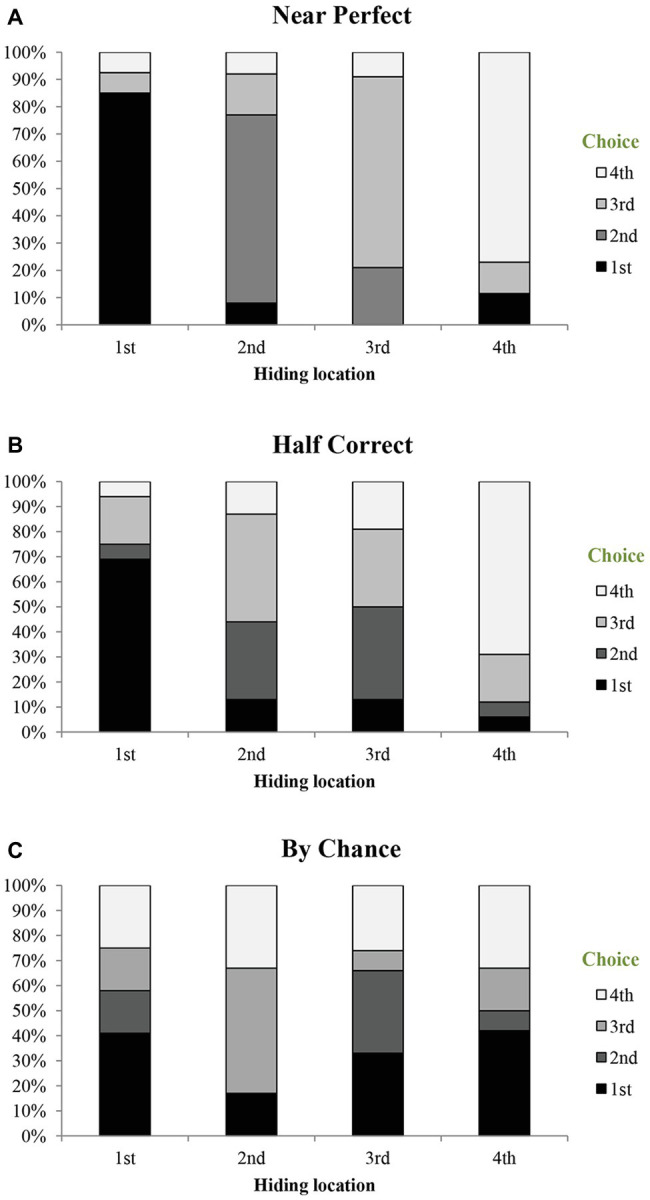
Percentage of location choices for each group in different conditions.

Next, we analyzed children’s incorrect choices by including only participants who had made at least one choice incorrectly. In other words, the Perfect group was excluded. We computed their choices in each condition of Hidden Position (see Figure 3), compared between these choices and compared the percentage of each choice to chance level (25%). The results showed a distinctive pattern of choices for the three groups.

For the Near Perfect group, we conducted four repeated measure tests for the four Hidden Position conditions, with position choice as independent variable. The tests found significant effects in all the four conditions, *Fs* > 13.64, *ps* < 0.001, and *η_p_* ^2^ *s* > 0.35. Multiple comparisons (Bonferroni) showed that children in this group chose the correct position significantly more often than the other three positions, regardless where the correct position was (*p* = 0.008 and *p* = 0.026 for the comparisons between position 2 and 3 when the coin was hidden in the second and third positions, and *ps* < 0.001 for all the other comparisons). The comparisons between each choice to chance level revealed the same pattern. Children in this group performed above chance in all of the four conditions, *ps* < 0.01. Furthermore, they successfully rejected all three incorrect railcars when the coin was hidden in either the first or last railcar, *ps* < 0.05. However, when the coin was hidden in the second railcar, they successfully rejected the first and last railcars, *ps* < 0.01, but not the third one, *t*(25) = −1.33, *p* = 0.20. When the coin was hidden in the third railcar, they also successfully rejected the first and last railcars, but not the second one, *t*(25) = −0.73, *p* = 0.47.

For the Half Correct group, similar repeated measure tests with position as independent variable found significant effects only when the coin was hidden in the first or last location, *Fs* > 8.22, *ps* < 0.001, and *η_p_* ^2^ *s* > 0.35. Multiple comparisons (Bonferroni) showed that when the coin was hidden in the first location, children chose this correct location more than the second and last location, *ps* < 0.01. And when the coin was hidden in the last location, children chose this correct location more than the first and second locations, *ps* < 0.01. However, when the coin was hidden in the middle two locations, this effect was not significant, *Fs* < 1.55, *ps* > 0.21. The comparison between each choice to chance level revealed the same pattern. Children in this group performed above chance when the coin was hidden in the first or last location, *ps* < 0.01, but not in the other two conditions. Their error pattern was also different from the Near Perfect group. When the coin was hidden in the first railcar, the children successfully rejected the second and the last railcars, *ps* < 0.05, but not the third one, *t*(15) = −0.62, *p* = 0.54. When the coin was hidden in the last railcar, they successfully rejected the first and second railcars, *ps* < 0.01, but not the third one, *t*(15) = −0.62, *p* = 0.54. When the coin was hidden in the second or third railcar, however, children did not reject any of the three incorrect railcars, *ps* > 0.05.

In contrast, children in the At Chance group chose all the four locations equally, regardless where the correct position was, *Fs* < 2.50, *ps* > 0.07. Additionally, they did not reject any of the incorrect railcars in all the conditions, *ps* > 0.05, which indicated that they chose by chance (see Table 2).

**Table 2:** Performance of each group in different conditions.

	First	Second	Third	Fourth
Near Perfect	P	P third	P second	P
Half Correct	P third	F	F	P third
At Chance	F	F	F	F

P, pass; F, fail. Numbers represent the locations children could not reject correctly.

## Discussion

To explore the representations that children use to encode locations, the present study modified the method used by Hu et al. (2017) by using four rather than two locations in a series. Specifically, we aimed at determining whether children use ordinal coding for these locations or a simple front-back representation. We found two key findings. First, although 3–5-year-olds performed above chance, they encountered different challenges when the target location was in different ordinal positions. Second, children with different performance levels demonstrated different error patterns. Together, these two facets of findings depict the different levels of the representations that the children used.

The first conclusion that we can draw from the findings is that most children in these age groups have difficulty using ordinal representation; therefore, the “front-back” array is one major helpful cue for them to encode the locations in the experiment. The results showed that children’s performance varied with the hiding locations. When the coin was hidden in the first or last location, children performed better than when it was hidden in the two middle locations. This indicated that at least a majority of children in the study used a front-back representation. They encoded the first location as “in the front,” the last as “in the back,” but had no label to encode the middle two. Additionally, children’s incorrect choices provided further evidence. When the coin was hidden in one of the two middle locations, children in the Near Perfect group successfully rejected the first and last locations, but could hardly discriminate between the middle two. In contrast, when the coin was hidden in the first or last locations, they rarely made mistakes. Therefore, we speculate that these children used a “front-middle-back” representation. They perceived the four locations as three parts: front, middle, and back. Therefore, they encoded the first and the last locations precisely but confused the middle two. The subsequent acquisition of using ordinal coding would help them to represent each of the four locations appropriately and distinctively. Twenty-three children had reached this stage and performed 100% correctly in our experiment (the Perfect group).

Some previous findings have provided support for the “front-middle-back” representation that occurs in this age. Children of 4 and 5 years of age are able to encode the middle location between two landmarks (Spetch and Parent, 2006; Uttal et al., 2006; Simms and Gentner, 2008). Although this achievement was observed in the frame of allocentric reference, in which the target location located between two salient landmarks, not between other alternative locations, it indicates that children can form a representation of “middle.” Furthermore, some other studies have provided evidence that children comprehend the word “between” in the third year (Johnston, 1988), and the verbal prompt of “middle” can improve the performance of three-and-a-half year-olds in position-mapping tasks (Loewenstein and Gentner, 2005). These findings indicate that children as young as 3 years can form a representation of spatial relations involving “middle” or “between.” Therefore, children in our study have the prerequisites to use a “front-middle-back” representation to resolve the task.

Furthermore, our results suggest salient individual differences in children’s development of spatial representation. Unlike in Hu et al. (2017), age difference was not evident in the current study. Instead, the error analysis revealed that error patterns may depend on their competence level in the tasks. It is children’s ability to parse a complex serial that influences their performance. As mentioned earlier, the Near Perfect group might have used a “front-middle-back” representation and thus could hardly discriminate between the middle two locations. Children with less competence, who succeeded in half of the trials, seemed to have no representation of “middle.” When the coin was hidden in one of the two middle locations, this group appeared to choose randomly among the four locations. In contrast, when the coin was hidden in the first or last location, they chose the correct location above chance and successfully rejected the location at the other end. Therefore, we speculate that these children had used a “front-back” representation, which appears to be not clear enough to encode specific locations. On the one hand, the overall serial might be roughly parsed into two halves, so the third location was not rejected correctly when the target was the last location. On the other hand, the two ends of the layout are more salient, leading to the successful rejection of the other end. Last, children with the least competence level in the experiment chose the locations randomly. Even a vague “front-back” representation to encode locations appeared to be too challenging for them. This variety in representation suggests that, when the task involves more complex and more diversified intrinsic relation information, children show significant individual differences in competence and therefore, form and use different representation. Interestingly, the individual differences in competence and representation do not correspond with the differences in age in months (see Table 1). Individual differences are commonly found in spatial tasks. Individuals with different spatial abilities construct spatial representations in different qualities and maintain the representations in different ways. These individual differences were mainly attributed to memory resources for storage and processing of spatial information (see a review by Hegarty and Waller, 2005). Future studies could assess children’s spatial working memory and its relationship with children’s choices of representations.

Additionally, future studies could perform more detailed analysis in the process of change in representation, such as using microgenetic method. Additionally, the selective using of various representations is another interesting issue. According to the model of strategy choice (Siegler and Taraban, 1986), children use diverse strategies simultaneously and make adaptive choices among them. Similarly, we speculate that the earlier-appeared representation will exist over prolonged periods of time, or even never faded out. That is, the children who used ordinal representation in this experiment could also use front-back representation in other situations, in which the front-back representation is more adaptive. Therefore, whether children used to choose among these various representations in specific spatial task requires future exploration.

These findings challenge again the previous conclusion that intrinsic reference frame could not be used in children until age 5 (Nardini et al., 2006; Negen and Nardini, 2015) and support the argument that simple spatial relations in the intrinsic layout could be used much earlier than researchers believed before (Hu et al., 2017). More importantly, these results suggest that children use various strategies to represent the spatial relations in the intrinsic frames in their development. A clearer depiction of the developmental trajectories and influential factors for these changing representations will be valuable and interesting for future research. For instance, the relation between spatial language and the using of intrinsic reference frame is in debate now (Nardini et al., 2006; Miller et al., 2016). As ordinal information is usually encoded verbally (at least in adults), the occurrence of precise ordinal representation might be dependent on the ability of using ordinal numbers. Future studies could test this hypothesis and explore the role of spatial language in the development of using various strategies to represent locations in intrinsic reference frame.

In summary, the current study provides evidence that children use a variety of representation to encode spatial locations in a series. With lower competence, they develop a vague front-back representation of the layout, in which the two ends are more salient but the middle cannot be encoded. With higher competence, a clearer “front-middle-back” representation is mainly used. Children can encode the locations in the two ends accurately but confuse the two locations in the middle. Finally, children with the highest competence use ordinal representation and can encode each location in the series precisely.

## Data Availability Statement

The datasets generated for this study are available on request to the corresponding author.

## Ethics Statement

The studies involving human participants were reviewed and approved by the Institutional Review Board of the Faculty of Psychology, Beijing Normal University. Written informed consent to participate in this study was provided by the participants’ legal guardian/next of kin.

## Author Contributions

QH developed the study and concepts. QH and YF created the design. YF performed the testing and data collection. YF and QH drafted the manuscript. YS provided revisions. All authors contributed to the article and approved the submitted version.

### Conflict of Interest

The authors declare that the research was conducted in the absence of any commercial or financial relationships that could be construed as a potential conflict of interest.
